# Structure of a quinolone-stabilized cleavage complex of topoisomerase IV from *Klebsiella pneumoniae* and comparison with a related *Streptococcus pneumoniae* complex

**DOI:** 10.1107/S2059798316001212

**Published:** 2016-03-24

**Authors:** Dennis A. Veselkov, Ivan Laponogov, Xiao-Su Pan, Jogitha Selvarajah, Galyna B. Skamrova, Arthur Branstrom, Jana Narasimhan, Josyula V. N. Vara Prasad, L. Mark Fisher, Mark R. Sanderson

**Affiliations:** a Randall Division of Cell and Molecular Biophysics, King’s College London, 3rd Floor, New Hunt’s House, Guy’s Campus, London SE1 1UL, England; b Cardiovascular and Cell Sciences Research Institute, St George’s, University of London, Cranmer Terrace, London SW17 0RE, England; c PTC Therapeutics Inc., 100 Corporate Court, South Plainfield, NJ 07080, USA

**Keywords:** *Klebsiella pneumoniae*, cleavage complex, quinolone, levofloxacin, topoisomerase IV, DNA binding, isomerase, isomerase–DNA complex, topoisomerases, Gram-negative complexes, X-ray crystallography, protein–DNA–drug complexes

## Abstract

*Klebsiella pneumoniae* is a Gram-negative bacterium that is responsible for a range of common infections, including pulmonary pneumonia, bloodstream infections and meningitis. Certain strains of *Klebsiella* have become highly resistant to antibiotics. Despite the vast amount of research carried out on this class of bacteria, the molecular structure of its topoisomerase IV, a type II topoisomerase essential for catalysing chromosomal segregation, had remained unknown. In this paper, the structure of its DNA-cleavage complex is reported at 3.35 Å resolution. The complex is comprised of ParC breakage-reunion and ParE TOPRIM domains of *K. pneumoniae* topoisomerase IV with DNA stabilized by levofloxacin, a broad-spectrum fluoroquinolone antimicrobial agent. This complex is compared with a similar complex from *Streptococcus pneumoniae*, which has recently been solved.

## Abstract 2

Crystal structures of the cleavage complexes of topoisomerase IV from Gram-negative (*K. pneumoniae*) and Gram-positive (*S. pneumoniae*) bacterial pathogens stabilized by the clinically important antibacterial drug levofloxacin are presented, analysed and compared. For *K. pneumoniae*, this is the first high-resolution cleavage complex structure to be reported.

## 1. Introduction   

*Klebsiella* is a genus belonging to the Enterobacteriaceae family of Gram-negative bacilli, which is divided into seven species with demonstrated similarities in DNA homology: *K. pneumoniae*, *K. ozaenae*, *K. rhinoscleromatis*, *K. oxytoca*, *K. planticola*, *K. terrigena* and *K. ornithinolytica*. *K. pneumoniae* is the most medically important species of the genus owing to its high resistance to antibiotics. Significant morbidity and mortality has been associated with an emerging, highly drug-resistant strain of *K. pneumoniae* characterized as producing the carbapenemase enzyme (KPC-producing bacteria; Nordmann *et al.*, 2009 ▸). The best therapeutic approach to KPC-producing organisms has yet to be defined. However, common treatments (based on *in vitro* susceptibility testing) are the polymyxins, tigecycline and, less frequently, aminoglycoside antibiotics (Arnold *et al.*, 2011 ▸). Another effective strategy involves the limited use of certain antimicrobials, specifically fluoroquinolones and cephalo­sporins (Gasink *et al.*, 2009 ▸). Several new antibiotics are under development for KPC producers. These include combinations of existing β-lactam antibiotics with new β-lactamase inhibitors able to circumvent KPC resistance. Neoglycosides are novel aminoglycosides that have activity against KPC-producing bacteria that are also being developed (Chen *et al.*, 2012 ▸).

Type II topoisomerase enzymes play important roles in prokaryotic and eukaryotic DNA replication, recombination and transcription (Drlica *et al.*, 2008 ▸; Laponogov *et al.*, 2013 ▸; Lee *et al.*, 2013 ▸; Nitiss, 2009*a* ▸,*b* ▸; Schoeffler & Berger, 2008 ▸; Sissi & Palumbo, 2009 ▸; Vos *et al.*, 2011 ▸; Wendorff *et al.*, 2012 ▸; Wu *et al.*, 2011 ▸, 2013 ▸). In bacteria, topoisomerase IV, a tetramer of two ParC and two ParE subunits, unlinks daughter chromosomes prior to cell division, whereas the related enzyme gyrase, a GyrA_2_GyrB_2_ tetramer, supercoils DNA and helps unwind DNA at replication forks. Both enzymes act *via* a double-strand DNA break involving a cleavage complex and are targets for quinolone antimicrobials that act by trapping these enzymes at the DNA-cleavage stage and preventing strand re-joining (Drlica *et al.*, 2008 ▸).

Levofloxacin is a broad-spectrum third-generation fluoro­quinolone antibiotic. It is active against Gram-positive and Gram-negative bacteria and functions by inhibiting gyrase and topoisomerase IV (Drlica & Zhao, 1997 ▸; Laponogov *et al.*, 2010 ▸). Acquiring a deep structural and functional understanding of the mode of action of fluoroquinolones (Tomašić & Mašič, 2014 ▸) and the development of new drugs targeted against topoisomerase IV and gyrase from a wide range of Gram-positive and Gram-negative pathogenic bacteria are highly active areas of current research directed at overcoming the vexed problem of drug resistance (Bax *et al.*, 2010 ▸; Chan *et al.*, 2015 ▸; Drlica *et al.*, 2014 ▸; Mutsaev *et al.*, 2014 ▸; Pommier, 2013 ▸; Srikannathasan *et al.*, 2015 ▸).

Here, we report the first three-dimensional X-ray structure of a *K. pneumoniae* topoisomerase IV ParC/ParE cleavage complex with DNA stabilized by levofloxacin. The crystal structure provides structural information on topoisomerase IV from *K. pneumoniae*, a pathogen for which drug resistance is a serious concern. The structure of the ParC/ParE–DNA–levofloxacin binding site highlights the details of the cleavage-complex assembly that are essential for the rational design of *Klebsiella* topoisomerase inhibitors.

## 2. Materials and methods   

### 2.1. Cloning, expression and purification of *K. pneumoniae* and *Streptococcus pneumoniae* ParC55/ParE30   

**Figure 1:**
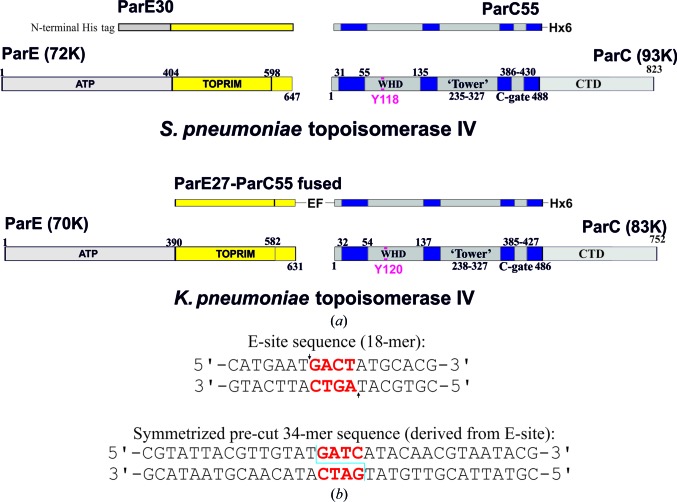
Protein and DNA used in the co-crystallization experiment. (*a*) Coloured diagram of the protein constructs used in crystallization. (*b*) DNA sequences used in crystallization. The 4 bp overhang is shown in red. Cleavage points are indicated by arrows.

Cloning, expression and purification protocols are described in detail in the Supporting Information. Table 1 ▸ contains the sequence information for all of the components of the complexes. Fig. 1 ▸ provides information about the protein and DNA constructs used in the experimental work.

### 2.2. Preparation of the DNA oligomer   

For the *K. pneumoniae* cleavage complex, two DNA oligomers (5′-CGTATTACGTTGTAT-3′ and 5′-GATCATACAACGTAATACG-3′) were synthesized by solid-phase phosphoramidite chemistry and doubly HPLC purified by Metabion, Munich, Germany. The DNA sequence was designed to make a complementary DNA 34-mer that contained the ‘pre-cut’ binding-site fragment: 5′-CGTATTACGTTGTAT↓GATCATACAACGTAATACG-3′ and 3′-GCATAATGCA­ACATACTAG↓TATGTTGCATTATGC-5′ (the cuts are shown by arrows; see Fig. 1 ▸ *b*).

For the *S. pneumoniae* cleavage complex, two DNA oligomers (5′-CATGAATGACTATGCACG-3′ and 5′-CGTGCATAGTCATTCATG-3′) were synthesized by solid-phase phosphoramidite chemistry and doubly HPLC purified by Metabion, Munich, Germany. The DNA sequence corresponds to the E-site 18-mer, which was found to be a better DNA length for crystallization of the *S. pneumoniae* topo­isomerase IV cleavage complexes in order to give stable reproducible crystals (see Fig. 1 ▸ *b*).

DNA stock solutions were made by mixing the required oligomers (at 1 m*M* in 20 m*M* Tris pH 7.5, 200 m*M* NaCl, 1 m*M* β-mercaptothanol, 0.05% NaN_3_) in equal volumes. For DNA annealing, the mixtures of complementary oligomers were heated to 98°C and then slowly cooled to 4°C over a 48 h period.

### 2.3. Crystallization and data collection   

Crystallization information for both the *S. pneumoniae* and the *K. pneumoniae* topoisomerase IV cleavage complexes is summarized in Table 2 ▸. Data-collection statistics and details are provided in Table 3 ▸. Structure-solution and refinement details are provided in Table 4 ▸.

#### 2.3.1. *S. pneumoniae* topoisomerase IV   

Protein was mixed with DNA in a 1:1:1.2 molar ratio (ParC55:ParE30:18-mer E-site DNA) with an overall concentration of 4 mg ml^−1^. Levofloxacin and magnesium chloride were added to final concentrations of 2 and 10 m*M*, respectively. The mixture was pre-incubated at room temperature overnight. Initial crystallization screening was performed by sitting-drop vapour diffusion in a 96-well MRC crystallization plate (600 nl protein mixture + 400 nl reservoir solution) using a Mosquito robot (TTP Labtech; http://www.ttplabtech.com). The best crystals were obtained using capillary counter-diffusion against 50 m*M* sodium cacodylate pH 6.5, 2.5% Tacsimate (Hampton Research; McPherson & Cudney, 2006 ▸), 7% 2-propanol, 62.5 m*M* KCl, 7.5 m*M* MgCl_2_ at 304 K. The crystals were flash-cooled at 100 K in cryoprotectant buffer *C* [50 m*M* sodium cacodyl­ate pH 6.5, 2.5% Tacsimate, 62.5 m*M* KCl, 7.5 m*M* MgCl_2_, 1 m*M* β-mercapto­ethanol, 30%(*v*/*v*) MPD]. The best data set was collected on beamline I03 at Diamond Light Source at a wavelength of 0.9763 Å using an ADSC Quantum 315 detector. The data extended to 2.6 Å resolution anisotropically and were used in refinement with a maximum-likelihood target in the initial refinement cycles; they were deposited in the PDB without introducing a resolution cutoff. However, owing to the high *R*_merge_ values in the outer shells, the final resolution is given as 2.9 Å and the statistics are reported according to this ‘trimmed’ resolution. The resolution cutoff was based on the rejection criteria *R*_merge_ < 50% and *I*/σ(*I*) > 1.5 in the highest resolution shell. The data were integrated using *HKL*-2000 (Otwinowski & Minor, 1997 ▸). The space group was determined to be *P*3_1_21, with unit-cell parameters *a* = *b* = 157.83, *c* = 211.15 Å.

The structure was solved by molecular replacement using *Phaser* (McCoy *et al.*, 2007 ▸) as implemented within the *CCP*4 suite (Winn *et al.*, 2011 ▸) and our previously published topo­isomerase IV–levofloxacin structure (PDB entry 3k9f; Laponogov *et al.*, 2010 ▸). Refinement was performed in *PHENIX* (Adams *et al.*, 2002 ▸, 2010 ▸) with manual inspection and corrections performed in *WinCoot* (Emsley & Cowtan, 2004 ▸; Emsley *et al.*, 2010 ▸).The structure was verified using *WinCoot* and *PROCHECK* (Laskowski *et al.*, 1993 ▸).

#### 2.3.2. *K. pneumoniae* topoisomerase IV   

ParC55/ParE30 protein stock in incubation buffer (at 4.5 mg ml^−1^) was mixed with the ‘pre-cut’ 34-mer DNA stock in a 1:1.2 protein:DNA molar ratio. High-concentration stocks of levofloxacin and MgCl_2_ were added to give final concentrations of 2 and 10 m*M*, respectively. The mixture was incubated overnight at room temperature. Initial crystallization screening was performed by sitting-drop vapour diffusion in 96-well MRC crystallization plates (600 nl protein mixture + 300 nl reservoir solution) using a Mosquito robot. When the optimal crystallization conditions had been established, conventional hanging-drop vapour diffusion in 24-well Linbro plates (4 µl protein mixture + 2 µl reservoir solution) was used to increase the crystal size.

Crystals formed after ∼7–10 d at room temperature. The crystallization conditions varied slightly from batch to batch in the range 0.1 *M* Tris pH 7.5–8.0, 0–50 m*M* NaCl, 4–8% PEG 4000, 12–15% glycerol.

It should be mentioned that several other DNA oligomers with the same binding-site sequence were tried for crystallization (*i.e.* 20-mer, ‘pre-cut’ 20-mer and 34-mer DNA sequences). However, these protein–DNA–drug complexes did not produce good-quality crystals for data collection.

Crystals were tested in-house for diffraction quality using an Oxford Xcalibur Nova CCD diffractometer and were then transported for high-resolution data collection at Diamond Light Source (Harwell Science and Innovation Campus, Oxfordshire, England). The data were collected on beamline I03 (wavelength 0.9762 Å) using a Pilatus 6M-F detector (0.2° oscillation per image, 100 K nitrogen stream). The best crystals diffracted to ∼3.2 Å resolution.

All data sets were integrated with *MOSFLM* (Leslie & Powell, 2007 ▸) and merged with *SCALA* (Evans, 2006 ▸) as implemented in *CCP*4 (Winn *et al.*, 2011 ▸). The ParC55/ParE30–DNA–levofloxacin crystals belonged to space group *P*2_1_, with unit-cell parameters *a* = 102.07, *b* = 161.53, *c* = 138.60 Å, α = 90.00, β = 94.22, γ = 90.00°. They contained two ParC/ParE–DNA heterodimers in the asymmetric unit.

Several data sets were collected, some of which contained visible diffraction to 3.2 Å resolution, but owing to potential internal twinning and space-group ambiguity (most data sets could be integrated in space groups *P*2_1_ and *P*2_1_2_1_2_1_) and the fact that the structure solution could be obtained in both space groups, careful selection of the integration ranges as well as appropriate data truncation were necessary. The best region of data was integrated to 3.35 Å (see Table 3 ▸ for statistics). The resolution cutoff was based on the rejection criteria *R*_merge_ < 50% and *I*/σ(*I*) > 1.5 in the highest resolution shell.

The structure was solved by the molecular-replacement method in *Phaser* (McCoy *et al.*, 2007 ▸) using the levofloxacin–DNA cleavage complex of topoisomerase IV from *S. pneumoniae* as a search model (PDB entry 3rae; ∼41.8% sequence identity). Refinement was performed in *PHENIX* (Adams *et al.*, 2002 ▸, 2010 ▸) using secondary-structure restraints derived by superposition of the *K. pneumoniae* ParC/ParE model with the previously solved complex of *S. pneumoniae* ParC/ParE. Rigid-body, positional and TLS refinements were performed. Levofloxacin molecules and magnesium ions were placed during the final stages of refinement based on missing electron density in the σ_A_-weighted 2*F*_obs_ − *F*_calc_ and *F*_obs_ − *F*_calc_ maps. *WinCoot* (Emsley & Cowtan, 2004 ▸) was used for interactive model fitting. The structure was verified using *WinCoot* and *PROCHECK* (Laskowski *et al.*, 1993 ▸). The resulting model had good geometry, with 87.8, 9.9 and 1.3% of residues in the favoured, allowed and generously allowed regions of the Ramachandran plot, respectively, and no more than 1% of residues in disallowed regions. The data-collection and final refinement statistics are given in Tables 3 ▸ and 4 ▸. Sequence alignment was performed in *ClustalW* (Larkin *et al.*, 2007 ▸, McWilliam *et al.*, 2013 ▸). Figures were prepared using *PyMOL* (DeLano, 2008 ▸), *CHEMDRAW* (Evans, 2014 ▸) and *CorelDRAW* (http://www.coreldraw.com).

## 3. Results and discussion   

We have co-crystallized the *K. pneumoniae* topoisomerase IV ParC/ParE breakage-reunion domain (ParC55; residues 1–490) and ParE TOPRIM domain (ParE30; residues 390–631) with a precut 34 bp DNA duplex (the E-site), stabilized by levofloxacin. The X-ray crystal structure of the complex was determined to 3.35 Å resolution, revealing a closed ParC55 dimer flanked by two ParE30 monomers (Figs. 1 ▸, 2 ▸ and 3 ▸). The overall architecture of this complex is similar to that found for *S. pneumoniae* topoisomerase–DNA–drug complexes (Laponogov *et al.*, 2009 ▸, 2010 ▸). Residues 6–30 of the N-terminal α-helix α1 of the ParC subunit again embrace the ParE subunit, ‘hugging’ the ParE subunits close to either side of the ParC dimer (Laponogov *et al.*, 2010 ▸). Deletion of this ‘arm’ α1 results in loss of DNA-cleavage activity (Laponogov *et al.*, 2007 ▸) and is clearly very important in complex stability (Fig. 3 ▸). This structural feature was absent in our original ParC55 structure (Laponogov *et al.*, 2007 ▸; Sohi *et al.*, 2008 ▸). The upper region of the topoisomerase complex consists of the E-subunit TOPRIM metal-binding domain formed of four parallel β-sheets and the surrounding α-helices. The C-subunit provides the WHD (winged-helix domain; a CAP-like structure; McKay & Steitz, 1981 ▸) and the ‘tower’ which form the U groove-shaped protein region into which the G-gate DNA binds with an induced U-shaped bend. The lower C-gate region (Fig. 3 ▸) consists of the same disposition of pairs of two long α-helices terminated by a spanning short α-helix forming a 30 Å wide DNA-accommodating cavity through which the T-gate DNA passes as found in the *S. pneumoniae* complex. Owing to the structural similarity, it appears that the topo­isomerases IV from *K. pneumoniae* and *S. pneumoniae* are likely to follow a similar overall topoisomerase catalytic cycle as shown in Fig. 4 ▸; we have confirmation of one intermediate from our recent structure of the full complex (the holoenzyme less the CTD β-pinwheel domain) with the ATPase domain in the open conformation (Laponogov *et al.*, 2013 ▸).

The G-gate DNA for the *S. pneumoniae* complex consists of an 18-base-pair E-site sequence (our designation for a DNA site which we first found from DNA-mapping studies; Leo *et al.*, 2005 ▸; Arnoldi *et al.*, 2013 ▸; Fig. 1 ▸). The crystallized complex was formed by turning over the topoisomerase tetramer in the presence of DNA and levofloxacin and crystallizing the product. In contrast, the *K. pneumoniae* complex was formed by co-crystallizing the topoisomerase tetramer complex in the presence of a 34-base-pair pre-cleaved DNA in the presence of levofloxacin. In both cases the DNA is bent into a U-form and bound snugly against the protein of the G-gate. We have been able to unambiguously read off the DNA sequences in the electron-density maps.

**Figure 2:**
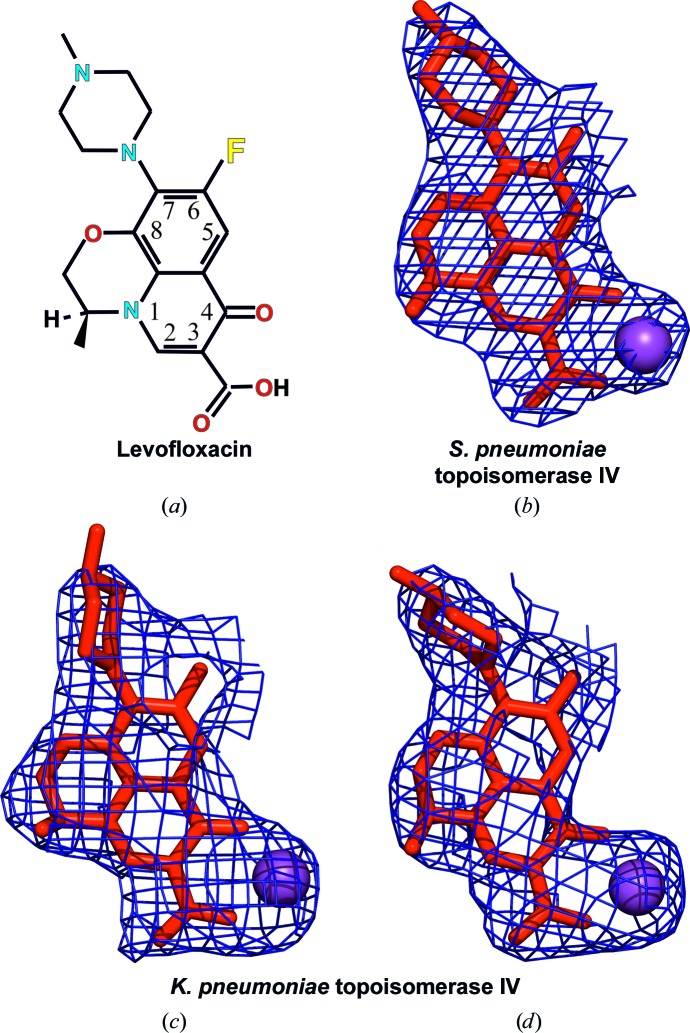
Chemical structure of levofloxacin (*a*) and its conformations observed within the active sites of *S. pneumoniae* topoisomerase IV (*b*) and *K. pneumoniae* topoisomerase IV (*c*, *d*). Electron-density maps (2*F*_obs_ − *F*_calc_) are shown as meshes for the drug molecules contoured at 1.5σ and are limited to a distance of 2.3 Å from the drug atoms.

**Figure 3:**
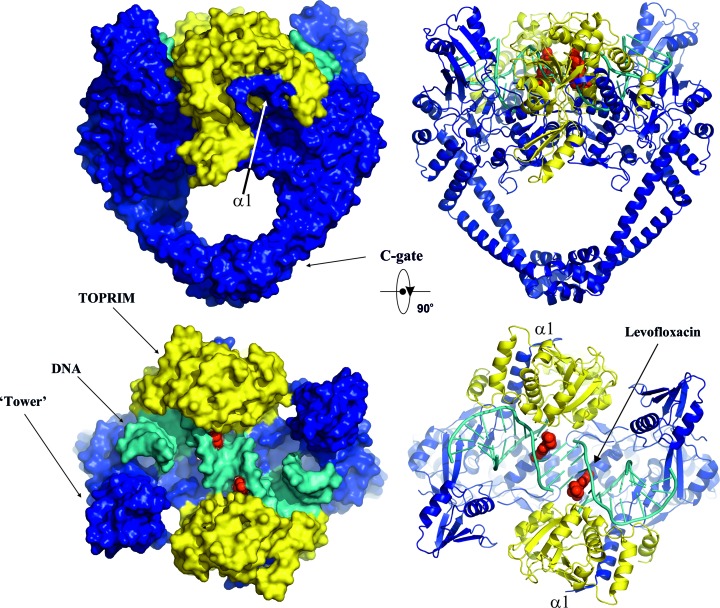
Overall orthogonal views of the cleavage complex of topoisomerase IV from *K. pneumoniae* in surface (left) and cartoon (right) representations. The ParC subunit is in blue, ParE is in yellow and DNA is in cyan. The bound quinolone molecules (levofloxacin) are in red and are shown using van der Waals representation.

**Figure 4:**
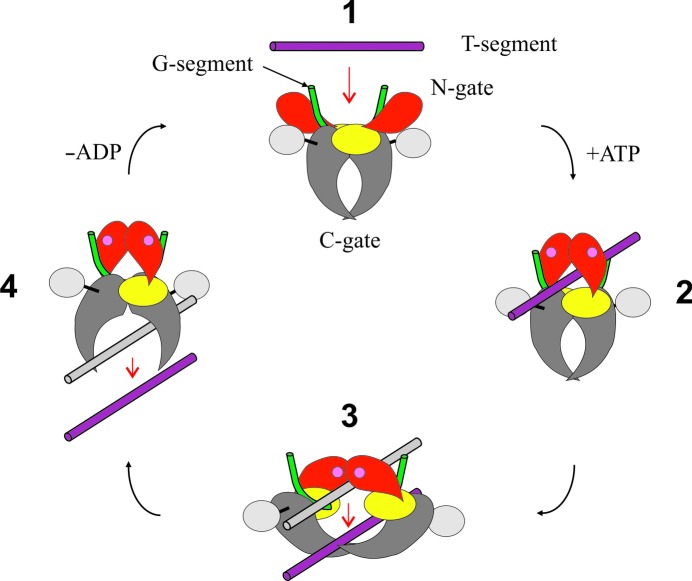
Schematic representation of the catalytic cycle of type II topoisomerases. The ParC N-terminal domain (ParC55) is in grey, the ParC C-terminal β-­pinwheel domain is in silver, the ParE N-terminal ATPase domain is in red, the ParE C-terminal domain (ParE30) is in yellow, the G-gate DNA is in green and the T-segment DNA is in purple. Bound ATP is indicated by pink circles in the ATPase domains (reproduced with permission from Fig. 1 of Lapanogov *et al.*, 2013).

**Figure 5:**
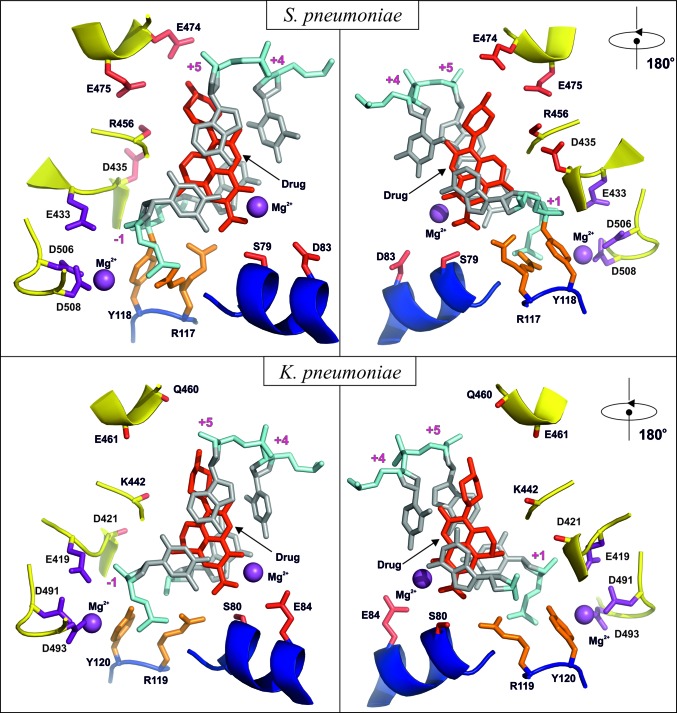
Detailed views of the active sites of topoisomerase IV from *S. pneumoniae* and *K. pneumoniae* with quinolone molecules bound. The magnesium ions and their coordinating amino acids are shown in purple. The drug molecules and residues known to lead to drug resistance upon mutation are in red. The active-site tyrosine and arginine are in orange. The DNA is shown in silver/cyan. The ParC and ParE backbones are shown in blue and yellow, respectively.

There is 41.6% sequence identity and 54.4% sequence homology between the ParE subunit of *K. pneumoniae* and that of *S. pneumoniae*. For the ParC subunits, the figures are 40.8 identity and 55.6% homology between the two organisms. The sequence alignment is given in Supplementary Fig. S1, with the key metal-binding residues and those which give rise to quinolone resistance highlighted. The binding of levofloxacin in the *K. pneumoniae* complex is shown in Figs. 2 ▸, 3 ▸ and 5 ▸ and is hemi-intercalated into the DNA and stacked against the DNA bases at the cleavage site (positions −1 and +1 of the four-base-pair staggered cut in the 34-mer DNA) which is similar to that found for the *S. pneumoniae* complex. Fig. 5 ▸ presents side-by-side views of the *K. pneumoniae* and *S. pneumoniae* active sites which shows that levofloxacin binds in a very similar manner in these two complexes with extensive π–π stacking interaction between the bases and the drug. The methylpiperazine at C7 (using the conventional quinolone numbering; C9 in the IUPAC numbering) on the drug extends towards residues Glu474 and Glu475 for *S. pneumoniae* and towards Gln460 and Glu461 for *K. pneumoniae*, where the glutamate at 474 is substituted by a glutamine at 460 in the *Klebsiella* strain. Interestingly, for *S. pneumoniae* we observe only one possible orientation of the C7 groups in both sub­units, while for *K. pneumoniae* we can see two: one with the same orientation as in *S. pneumoniae* and other rotated 180° away. They both exist within the same crystal in the two different dimers in the asymmetric unit. The side chains surrounding them in ParE are quite disordered and are more defined in *K. pneumoniae* (even though this complex is at lower resolution) than in *S. pneumoniae*. There are no direct hydrogen bonds from the drug to these residues (although it is possible that some are formed through water, which cannot be observed at this resolution). Obviously, the drug–ParE interaction in this region is less strong compared with PD 0305970 binding to the *S. pneumoniae* DNA complex, where PD 0305970 forms a hydrogen bond to ParE residue Asp475 and can form one to Asp474 if the bond rotates (Laponogov *et al.*, 2010 ▸). This may explain why drug-resistance mutations for levofloxacin are more likely to form in the ParC subunits rather than in the ParE subunits.

For both complexes there is a Mg^2+^ ion bound to levofloxacin between the carbonyl group at position 4 of the quinolone and the carboxyl at position 6 (Figs. 2 ▸ and 5 ▸ and Supplementary Fig. 2 ▸). For *S. pneumoniae* topoisomerase IV, one of the O atoms of the carboxyl of Asp83 points towards the Mg^2+^ ion and is within hydrogen-bonding distance (5.04 Å) through an Mg^2+^-coordinated water. For *K. pneumoniae* both of the carboxyl O atoms are pointing towards the Mg^2+^ ion at distances of 4.86 and 4.23 Å. These residues are ordered in only one of the two dimers in the *K. pneumoniae* crystal (the one in which the C7 group is pointing towards the DNA away from ParE, although the conformations of these two groups on the drug are probably not correlated).

**Figure 6:**
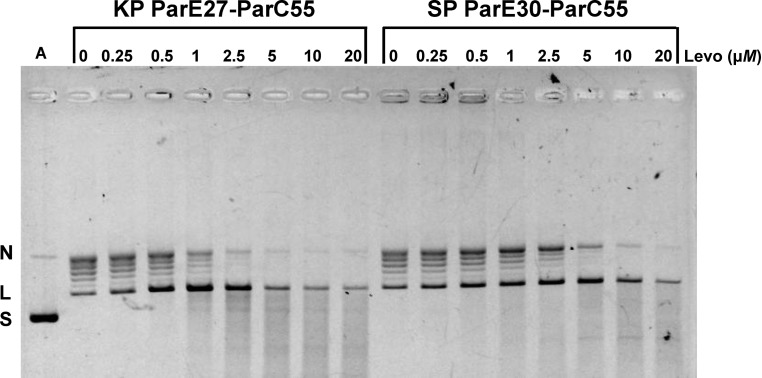
Comparison of DNA cleavage by topoisomerase IV core ParE-ParC fusion proteins from *K. pneumoniae* (KP) and *S. pneumoniae* (SP) promoted by levofloxacin. Supercoiled plasmid pBR322 (400 ng) was incubated with topoisomerase IV proteins (400 ng) in the absence or presence of levofloxacin at the indicated concentrations. After 60 min incubation, samples were treated with SDS and proteinase K to remove proteins covalent bound to DNA, and the DNA products were examined by gel electrophoresis in 1% agarose. Lane A, supercoiled pBR322 DNA; N, L and S, nicked, linear and supercoiled pBR322, respectively.

The topoisomerase IV ParE27-ParC55 fusion protein from *K. pneumoniae* was fully active in promoting levofloxacin-mediated cleavage of DNA (Fig. 6 ▸). In the absence of the drug and ATP, the protein converted supercoiled pBR322 into a ladder of bands corresponding to relaxed DNA. The inclusion of levofloxacin produced linear DNA in a dose-dependent and ATP-independent fashion. Similar behaviour was observed for the *S. pneumoniae* topo­isomerase IV ParE30-ParC55 fusion protein. The CC_25_ (the drug concentration that converted 25% of the supercoiled DNA substrate to a linear form) was 0.5 µ*M* for the *Klebsiella* enzyme and 1 µ*M* for the pneumococcal enzyme. Interestingly, *K. pneumoniae* strains are much more susceptible to levofloxacin than *S. pneumoniae*, with typical MIC values of 0.016 and 1 mg l^−1^, respectively (Odenholt & Cars, 2006 ▸), reflecting differences in multiple factors (in addition to binding affinity) that influence drug responses, including membrane, peptidoglycan structure, drug-uptake and efflux mechanisms. Moreover, although topoisomerase IV is primarily the target of levofloxacin in *S. pneumoniae*, it is likely to be gyrase in the Gram-negative *K. pneumoniae*.

In summary, we have determined the first structure of a quinolone–DNA cleavage complex involving a type II topo­isomerase from *K. pneumoniae*. Given the current concerns about drug-resistant strains of *Klebsiella*, the structure reported here provides key information in understanding the action of currently used quinolones and should aid in the development of other topoisomerase-targeting therapeutics active against this major human pathogen.

## Supplementary Material

PDB reference: topoisomerase IV cleavage complex from *Klebsiella pneumoniae*, 5eix

PDB reference: from *Streptococcus pneumoniae*, 3rae

Supporting Information.. DOI: 10.1107/S2059798316001212/mn5108sup1.pdf

## Figures and Tables

**(a):** *K. pneumoniae* topoisomerase IV.

Source organism	*K. pneumoniae* (strain ATCC 35657)
Expression vector	pET-29a
Expression host	*E. coli* BL21(λDE3) pLysS
Complete amino-acid sequence of the construct produced	
Topoisomerase IV (ParE CTD 390–631 and ParC NTD 1–490 fused)	MKKLTSGPALPGKLADCTAQDLNRTELFLVEGDSAGGSAKQARDREYQAIMPLKGKILNTWEVSSDEVLASQEVHDISVAIGIDPDSDDLSQLRYGKICILADADSDGLHIATLLCALFVRHFRTLVKEGHVYVALPPLYRIDLGKEVYYALTEEEKTGVLEQLKRKKGKPNVQRFKGLGEMNPMQLRETTLDPNTRRLVQLVISDEDEQQTTAIMDMLLAKKRSEDRRNWLQEKGDMADLEVEFMSDMAERLALHEFTENAYLNYSMYVIMDRALPFIGDGLKPVQRRIVYAMSELGLNASAKFKKSARTVGDVLGKYHPHGDSACYEAMVLMAQPFSYRYPLGDGQGNWGAPDDPKSFAAMRYTESRLSKYAELLLSELGQGTVDWVPNFDGTLQEPKMLPARLPNILLNGTTGIAVGMATDIPPHNLREVAKAAITLIEQPKTTLDELLDIVQGPDFPTEAEIITSRAEIRKIYQNGRGSVRMRAVWSKEDGAVVITALPHQVSGAKVLEQIAAQMRNKKLPMVDDLRDESDHENPTRLVIVPRSNRVDMEQVMNHLFATTDLEKSYRINLNMIGLDGRPAVKNLLEILSEWLVFRRDTVRRRLNHRLEKVLKRLHILEGLLVAFLNIDEVIEIIRTEDEPKPALMSRFGISETQAEAILELKLRHLAKLEEMKIRGEQSELEKERDQLQAILASERKMNNLLKKELQADADAFGDDRRSPLHEREEAKAMSHHHHHH
Symmetrized E-site (pre-cut) DNA1	5′-CGTATTACGTTGTAT-3′
Symmetrized E-site (pre-cut) DNA2	5′-GATCATACAACGTAATACG-3′

**(b):** *S. pneumoniae* topoisomerase IV.

Source organism	*S. pneumoniae* (isolate 7785 St George’s Hospital; Pan & Fisher, 1996 ▸)
Expression vector	pET-19b (N-terminal His_10_), pET-29a (C-terminal His_6_)
Expression host	*E. coli* BL21(λDE3) pLysS
Complete amino-acid sequence of the construct produced	
ParC55	MSNIQNMSLEDIMGERFGRYSKYIIQDRALPDIRDGLKPVQRRILYSMNKDSNTFDKSYRKSAKSVGNIMGNFHPHGDSSIYDAMVRMSQNWKNREILVEMHGNNGSMDGDPPAAMRYTEARLSEIAGYLLQDIEKKTVPFAWNFDDTEKEPTVLPAAFPNLLVNGSTGISAGYATDIPPHNLAEVIDAAVYMIDHPTAKIDKLMEFLPGPDFPTGAIIQGRDEIKKAYETGKGRVVVRSKTEIEKLKGGKEQIVITEIPYEINKANLVKKIDDVRVNNKVAGIAEVRDESDRDGLRIAIELKKDANTELVLNYLFKYTDLQINYNFNMVAIDNFTPRQVGIVPILSSYIAHRREVILARSRFDKEKAEKRLHIVEGLIRVISILDEVIALIRASENKADAKENLKVSYDFTEEQAEAIVTLQLYRLTNTDVVVLQEEEAELREKIAMLAAIIGDERTMYNLMKKELREVKKKFATPRLSSLEDTAKALEHHHHHH
ParE30	MGHHHHHHHHHHSSGHIDDDDKHMKNKKDKGLLSGKLTPAQSKNPAKNELYLVEGDSAGGSAKQGRDRKFQAILPLRGKVINTAKAKMADILKNEEINTMIYTIGAGVGADFSIEDANYDKIIIMTDADTDGAHIQTLLLTFFYRYMRPLVEAGHVYIALPPLYKMSKGKGKKEEVAYAWTDGELEELRKQFGKGATLQRYKGLGEMNADQLWETTMNPETRTLIRVTIEDLARAERRVNVLMGDKVEPRRKWIEDNVKFTLEEATVF
E-site DNA1	5′-CATGAATGACTATGCACG-3′
E-site DNA2	5′-CGTGCATAGTCATTCATG-3′

**Table 2:** Crystallization

	*K. pneumoniae* topoisomerase IV	*S. pneumoniae* topoisomerase IV
Method	Vapour diffusion	Capillary counter-diffusion
Plate type	24-well Limbro	N/A
Temperature (K)	298	304
Protein concentration (mg ml^−1^)	4.5	4
Buffer composition of protein solution	20 m*M* Tris pH 7.5, 100 m*M* NaCl, 1 m*M* β-mercaptoethanol, 0.05% NaN_3_	
Composition of reservoir solution	0.1 *M* Tris pH 7.5–8.0, 0–50 m*M* NaCl, 4–8% PEG 4000, 12–15% glycerol	50 m*M* sodium cacodylate pH 6.5, 2.5% Tacsimate, 7% 2-propanol, 62.5 m*M* KCl, 7.5 m*M* MgCl_2_
Volume and ratio of drop	4 + 2 µl	N/A
Volume of reservoir (ml)	0.5	N/A

**Table 3:** Data collection and processing Values in parentheses are for the outer shell.

	*K. pneumoniae* topoisomerase IV	*S. pneumoniae* topoisomerase IV
Diffraction source	Beamline I03, Diamond Light Source	
Wavelength (Å)	0.97620	0.97630
Temperature (K)	100.0	100.0
Detector	Pilatus 6M-F	ADSC Quantum 315
Crystal-to-detector distance (mm)	502.22	377.629
Rotation range per image (°)	0.2	0.25
Total rotation range (°)	180	75
Exposure time per image (s)	0.2	1.0
Space group	*P*2_1_	*P*3_1_21
*a*, *b*, *c* (Å)	102.07, 161.53, 138.60	157.83, 157.83, 211.15
α, β, γ (°)	90, 94.22, 90	
Mosaicity (°)	0.237	0.466
Resolution range (Å)	86.12–3.35 (3.53–3.35)	50–2.90 (3.00–2.90)
Total No. of reflections	160764	311576
No. of unique reflections	63406	67471
Completeness (%)	98.5 (98.4)	99.4 (99.9)
Multiplicity	2.53 (2.59)	4.6 (4.7)
〈*I*/σ(*I*)〉	3.48 (1.95)	16.14 (3.48)
*R*_r.i.m._ †	0.116 (0.434)	0.08 (0.515)
Overall *B* factor from Wilson plot (Å^2^)	53.29	73.37

† Estimated *R*_r.i.m._ = *R*_merge_[*N*/(*N* − 1)]^1/2^, where *N* is the data multiplicity.

**Table 4:** Structure solution and refinement Values in parentheses are for the outer shell.

	*K. pneumoniae* topoisomerase IV	*S. pneumoniae* topoisomerase IV
Resolution range (Å)	85.01–3.35 (3.40–3.35)	41.83–2.90 (2.93–2.90)
Completeness (%)	98.3	99.5
σ Cutoff	*F* > 1.350σ(*F*)	*F* > 1.34σ(*F*)
No. of reflections, working set	60158 (2615)	67471 (1992)
No. of reflections, test set	3208 (142)	6838 (218)
Final *R*_cryst_	0.224 (0.2990)	0.186 (0.2806)
Final *R*_free_	0.259 (0.3537)	0.226 (0.3562)
No. of non-H atoms		
Protein	18741	10338
Nucleic acid	1608	730
Ligand	104	52
Ion	8	6
Water	—	54
Total	20461	11180
R.m.s. deviations		
Bonds (Å)	0.002	0.008
Angles (°)	0.611	1.221
Average *B* factors (Å^2^)		
Protein	58.05	76.7
Nucleic acid	64.85	90.7
Ligand	60.14	95.7
Ion	42.62	84.5
Water	—	64.2
Ramachandran plot		
Most favoured (%)	93	94
Allowed (%)	6	6
