# Supplementary material for: Structure of a quinolone-stabilized cleavage complex of topoisomerase IV from Klebsiella pneumoniae and comparison with a related Streptococcus pneumoniae complex
Source: Acta Crystallogr D Struct Biol. 2016 Mar 24;72(Pt 4):488–96. doi: 10.1107/S2059798316001212 (PMC4822561; doi:10.1107/S2059798316001212)
Supplement: Supplementary file 1 [file d-72-00488-sup1.pdf]

STRUCTURAL
BIOLOGY

Volume 72 (2016)

Supporting information for article:

Structure solution of a quinolone-stabilized cleavage complex of topoisomerase IV from *Klebsiella pneumoniae* and comparison with a related *Streptococcus pneumoniae* complex

Dennis A. Veselkov, Ivan Laponogov, Xiao-Su Pan, Jogitha Selvarajah, Galyna B. Skamrova, Arthur Branstrom, Jana Narasimhan, Josyula V. N. Vara Prasad, L. Mark Fisher and Mark R. Sanderson

S1. Materials and methods: cloning, expression and purification of *K. pneumoniae* and *S. pneumoniae* ParC55/ParE30

S1.1. *K. pneumoniae* topoisomerase IV

DNA oligomers for use in PCR were synthesized by Metabion International AG, Munich. The *parE30* fragment was amplified with forward primer Kp-E30K-NdeI-F (5'-AAA AAA CAT ATG AAA AAG CTG ACC AGC GGT-3') (nucleotide position 1186 to 1203 of the ParE gene occurs downstream of the underlined NdeI restriction site) and reverse primer Kp-E-EcoRI-R 5'-AAA AAA GAA TTC CAC TTC CAG ATC CGC CAT-3' includes nucleotide position 2341 towards the end of the *parE* gene). The EcoRI site introduced for cloning purposes in the reverse primer adds extra Glu and Phe residues that override the ParE stop codon.

DNA amplification was performed using Vent DNA polymerase with 1.5 mM $MgCl_2$. Genomic DNA from *K. pneumoniae* ATCC 35657 was used as template. PCR conditions were as follows: initial denaturing at 95° C for 2 minutes followed by 30 cycles of denaturing at 94° C for 45 sec, annealing at 58° C for 45 sec, and DNA polymerisation at 74° C for 2 min. The expected PCR product was a 740 bp fragment which was subsequently digested with NdeI and EcoRI, purified from agarose gel and ligated with similarly digested vector pET29a. The resulting expression plasmid was sequenced and the correctness of the reading frame was confirmed.

ParC55, encoded by a 1480 bp fragment, was amplified by PCR using forward primer Kp-C-EcoRI-F, 5'-AAA AAA GAA TTC ATG AGC GAT ATG GCA GAG-3' (EcoRI site just before the ATG start codon of parC gene) and reverse primer Kp-C55K-XhoI-R

5'-AAA AAA CTC GAG GCT CAT CGC TTT CGC TTC-3' (XhoI site immediately followed by the nucleotide 1470 of the *parC* gene, in frame for cloning into pET29a for C-terminal 6x His tag). PCR conditions were the same as that of the *parE30* described above.

Fusion protein was prepared by joining the *parE30-parC55* gene segment into an expression plasmid pET29a. The *parC55* PCR product was digested with EcoRI/XhoI, in addition to the plasmid that carried the *parE30* fragment. DNA fragments were purified from gels and ligated to link the *parE30* with *parC55*.

Plasmid was transformed into *E. coli* BL21(λDE3)pLysS. A five mL culture of the overnight growth was used to inoculate 750 mL of LB medium containing kanamycin (34 μg/mL). Cells were grown at 37 ^{o}C for approximately 3 hours until the optical density at 600 nm reached 0.4. Cells were then placed at 16 ^{o}C on an orbital shaker for 1 hour, before adding IPTG to 0.4 mM. The culture was allowed to incubate overnight for 16 hours. Cells were harvested by centrifugation, supernatant discarded, and the bacterial pellet resuspended in lysis buffer (20 mM Tris.HCl pH 8.0, 200 mM NaCl,

20 mM imidazole, 10% glycerol), and stored at -80 ºC. Six litres of cells in LB were induced and collected.

Cells were lysed by sonication and addition of lysozyme, supernatant from 120 ml of cell lysate was recovered by centrifugation and mixed with 2.5 ml of Ni-NTA at 4 ºC for 2 hours. The mixture was then loaded onto a column, and followed by successively washing and eluting using buffer containing increased imidazole concentration (40 mM, 100 mM, and 200 mM). Fractions containing the protein were pooled and 70 mg of fusion protein E30-C55 was recovered from 6 L culture.

S1.2. *S. pneumoniae* topoisomerase IV

S. pneumoniae ParC55 (residues 1–488) and ParE30 (residues 404–647) (Figure 1A) were produced as the C-terminally and N-terminally His-tagged proteins by over-expression in *E. coli* and purified as described previously (Laponogov *et al.*, 2010, Laponogov *et al.*, 2009, Laponogov *et al.*, 2007).

Prior to crystallization, the purified protein stocks were dialysed at 4ºC into incubation buffer (20 mM Tris pH 7.5, 100 mM NaCl, 1 mM β-mercaptoethanol and 0.05% NaN_3). For *S. pneumoniae* topoisomerase IV, ParC55 and ParE30 were first dialysed against the higher salt buffer (20 mM Tris pH 7.5, 200 mM NaCl, 1 mM β-mercptoethanol and 0.05% NaN_3) and then mixed at 1:1 molar ratio prior to dialysis into the final incubation buffer.

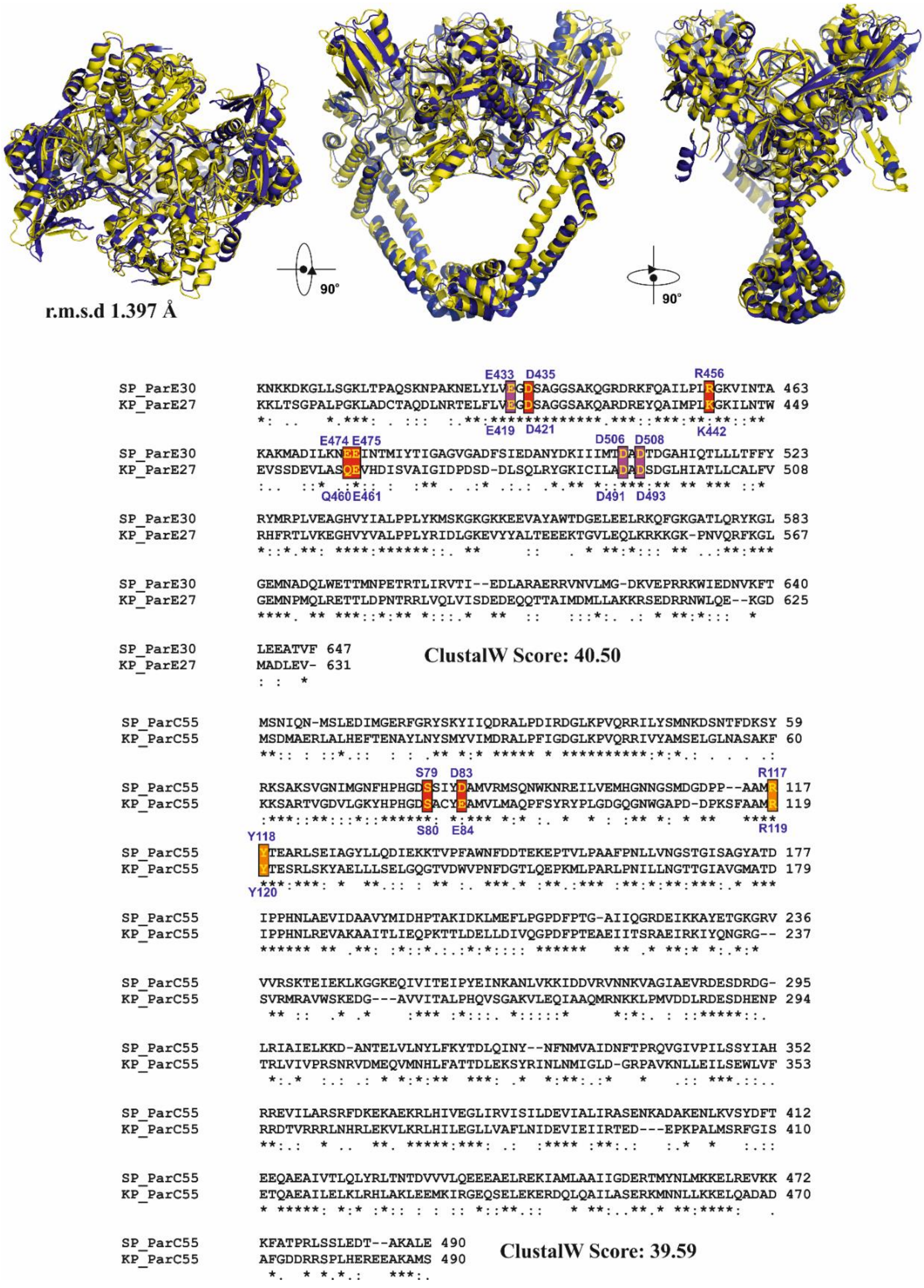

Figure S1 Overall superposition and sequence alignment of the cleavage complexes of topoisomerase IV from *S. pneumoniae* (blue) and *K. pneumoniae* (yellow). R.m.s.d. was calculated using the SSM function in WinCoot. Sequence alignment was done in ClustalW. Corresponding amino acids of interest are highlighted in the sequence: the magnesium ion-coordinating amino acids are shown in purple, the residues known to lead to drug-resistance upon mutation are in red, the active site tyrosine and arginine are in orange.

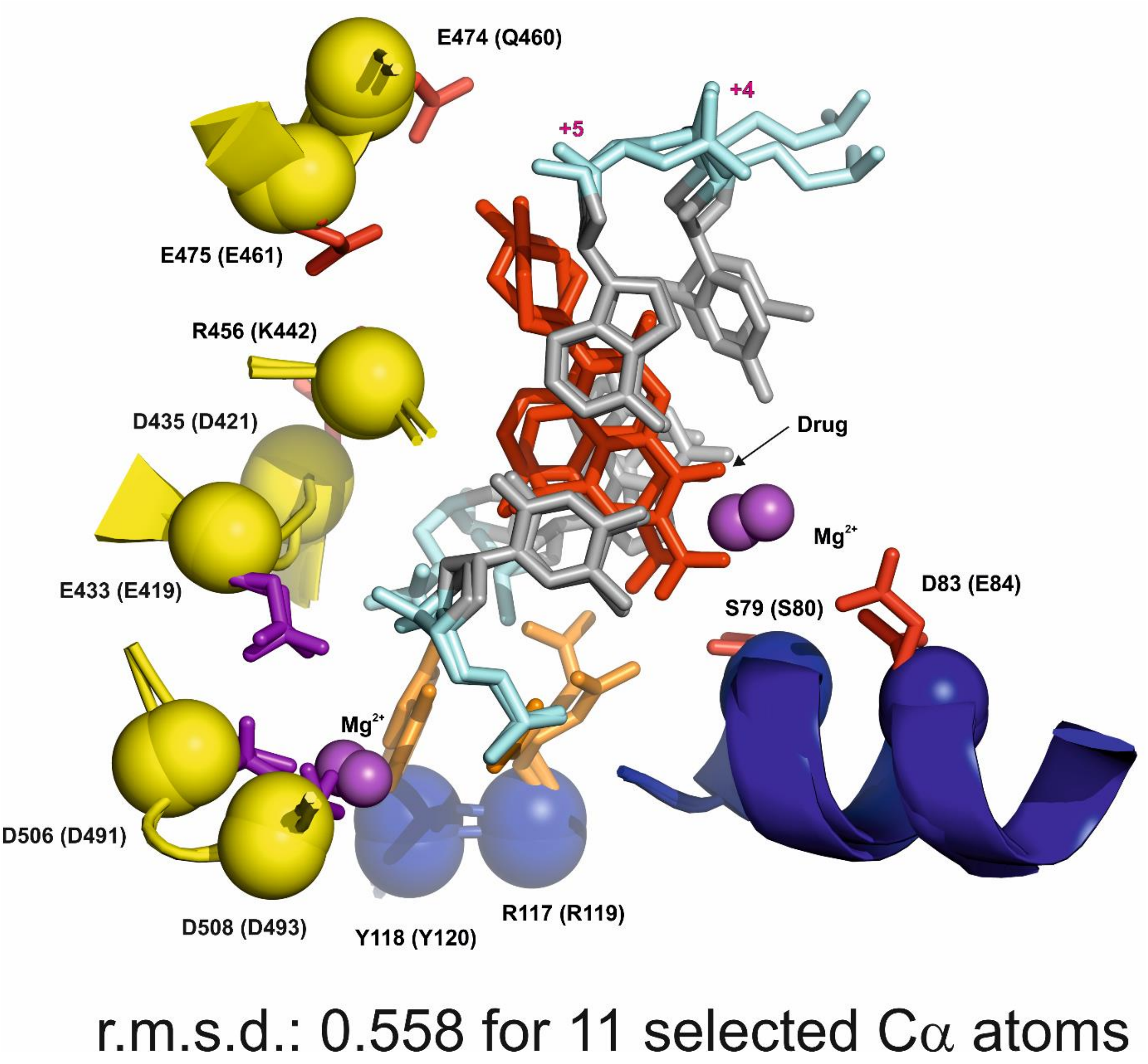

Figure S2 Superposition of the active sites of topoisomerase IV from *S. pneumoniae* and *K. pneumoniae* using only the selected Cα-s as the anchoring points. The superposition is performed in PyMOL and the corresponding r.m.s.d. for the selected Cα-s is 0.558 Å. The amino acid names and numbers are given for *S. pneumoniae* and *K. pneumoniae* (the latter are provided in brackets). The magnesium ions and their coordinating amino acid are shown in purple. The drug molecules and residues known to lead to drug-resistance upon mutation are in red. The active site tyrosine and arginine are in orange. The DNA is shown in silver/cyan. ParC and ParE backbones are shown in blue and yellow correspondingly. Cα-s used for the superposition least square fitting are indicated by large spheres.
